# Identifying neoantigens for use in immunotherapy

**DOI:** 10.1007/s00335-018-9771-6

**Published:** 2018-08-24

**Authors:** Sharon Hutchison, Antonia L. Pritchard

**Affiliations:** Genetics and Immunology Research Group, University of the Highlands and Islands, An Lòchran, 10 Inverness Campus, Inverness, IV2 5NA Scotland, UK

## Abstract

This review focuses on the types of cancer antigens that can be recognised by the immune system and form due to alterations in the cancer genome, including cancer testis, overexpressed and neoantigens. Specifically, neoantigens can form when cancer cell-specific mutations occur that result in alterations of the protein from ‘self’. This type of antigen can result in an immune response sufficient to clear tumour cells when activated. Furthermore, studies have reported that the likelihood of successful immunotherapeutic targeting of cancer by many different methods was reliant on immune response to neoantigens. The recent resurgence of interest in the immune response to tumour cells, in conjunction with technological advances, has resulted in a large increase in the predicted, identified and functionally confirmed neoantigens. This growth in identified neoantigen sequences has increased the contents of training sets for algorithms, which in turn improves the prediction of which genetic mutations may form neoantigens. Additionally, algorithms predicting how proteins will be processed into peptide epitopes by the proteasome and which peptides bind to the transporter complex are also improving with this research. Now that large screens of all the tumour-specific protein altering mutations are possible, the emerging data from assessment of the immunogenicity of neoantigens suggest that only a minority of variants will form targetable epitopes. The potential for immunotherapeutic targeting of neoantigens will therefore be greater in cancers with a higher frequency of protein altering somatic variants. There is considerable potential in the use of neoantigens to treat patients, either alone or in combination with other immunotherapies and with continued advancements, these potentials will be realised.

## Introduction

While the immune system has been known to play a role in the control of tumourigenic cells since the start of the twentieth century (historical review: Strebhardt and Ullrich 2008), research has yet to identify consistent methods to manipulate it to clear tumour cells. Significant technological advances have allowed researchers to molecularly characterise tumours and responding immune cells, which has resulted in breakthroughs that have translated to pharmacologically actionable markers and targets (as reviewed in Pritchard 2018). In order to reduce potential side effects to the patient, markers that are unique to cancer cells are particularly desirable. This review will focus on the immunogenic antigens that are expressed by cancer cells, the methods by which a particular type of antigen can be identified from genomic data and what is understood about the immunogenic potential of these antigens. Immune cells that can recognise cancer cells displaying antigen markers that are specific to tumour cells include CD4^+^ and CD8^+^ T-cells and B-cell subsets. This review only examines the immune targets displayed by cancer cells that are recognised by T-cells; a recommended review of the role of B-cells is Yuen et al. (2016).

## Immune recognition of antigens

In order to be displayed on cells, these antigens have to go through a process of protein cleavage and binding to MHC molecules; then in order to be recognised by the T-cell, a T-cell receptor (TCR) capable of binding the displayed peptide/MHC complex (pMHC) must be present. These processes are outlined in the following section.

### The major histocompatibility complex

The MHC (major histocompatibility complex) is expressed nearly ubiquitously on the majority of cells in vertebrates. The MHC displays protein fragments sampled from both within and outside the cell to alert the immune system to infection by pathogens. As the MHC display peptides from all protein sources, the T-cells recognising the MHC/peptide complexes have to be able to distinguish ‘self’ from ‘non-self’ to avoid autoimmunity. In humans, the HLA proteins are encoded by genes that form a cluster on chromosome 6. They are broadly split into two types: MHC-class I and MHC-class II molecules. Humans have three classical MHC-class I genes, called HLA-A, HLA-B and HLA-C, and three classical MHC-class II molecules: HLA-DR, HLA-DQ and HLA-DP; non-classical MHC molecules also exist. Different subtypes of T-cells recognise MHC/peptide complexes, with CD8^+^ T-cells recognising internally derived peptides bound to MHC-class I and CD4^+^ T-cells recognising peptides derived from external proteins bound to MHC-class II. A recommended comprehensive review on the structure of the MHC molecules is Blum et al. (2013).

### Protein processing by the proteasome

Proteins are processed into peptide fragments by the proteasome (Uebel and Tampe 1999). There are different proteasomes that can generate peptides for MHC-class I presentation, dependent on the cell source (Basler et al. 2013; Kloetzel 2004). The manner by which proteins are cleaved to form short peptides capable of binding to the MHC molecules has been examined. This has largely been based on in-depth assessment of the processing of specific proteins [e.g. enolase by the immunoproteasome (Toes et al. 2001) and β-casein by the 26S proteasome (Emmerich et al. 2000)] and by examining the known peptides produced and bound to MHC molecules, in the context of the whole protein. Despite the relatively few studies examining how whole proteins are processed through the different proteasomes, these data have been used as training sets for development of in silico prediction tools such as NetChop (Kesmir et al. 2002), BP-NN (Wang et al. 2013) and mhc-pathway (Tenzer et al. 2005).

### Peptide binding to MHC molecules

After peptides have been processed in the cytosol by the proteasome, they are selected for movement to the endoplasmic reticulum (ER) by their ability to bind the TAP (transporter associated with antigen processing) complex (Lehnert and Tampe 2017). The ability of the peptide to bind to TAP is also an aspect of MHC processing that has been investigated for predictability testing (e.g. Bhasin et al. 2007; Peters et al. 2003; Tenzer et al. 2005; Zhang et al. 2006). Once within the ER, peptides are loaded on to the HLA proteins based on their ability to fit in the binding groove. The HLA/peptide complex is then shuttled to the cell surface in complex with chaperone proteins for display to the immune system.

The nature of the binding of peptides to the MHC molecules and factors that can influence these interactions are discussed in the next sections; for more comprehensive reviews on the structure of the MHC molecules and recognition of the MHC-peptide complex by T-cells, please see Blum et al. (2013) and Wucherpfennig et al. (2010), respectively.

#### Peptide binding to MHC-class I

**Fig. 1:**
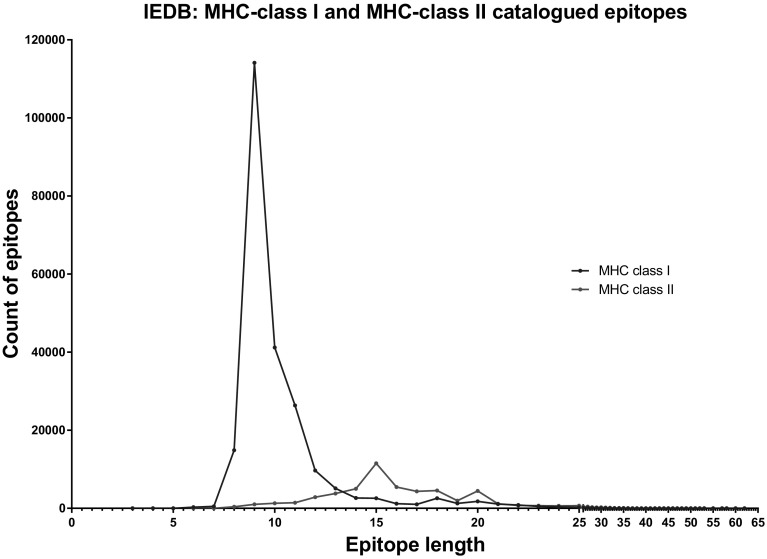
Total number of unique catalogued MHC-class I and MHC-class II peptides in the Immune Epitope Database (IEDB). Graph depicts the total number of unique peptides of each length stored in the database; the total number of MHC-class I peptides are 229,036 and MHC-class II are 54,607

X-ray crystallographic structure of the MHC-class I molecule showed that the binding groove is composed of two α-helical regions forming the sides and eight antiparallel β-strands that form its floor (Bjorkman et al. 1987a, b). MHC-class I molecules bind short peptide epitopes, with the N- and C-terminal ends being anchored into pockets at each end of the peptide binding groove (Natarajan et al. 1999). The different MHC-class I HLA subtypes tend to bind specific amino acids in these anchor points. The majority of MHC-class I binding peptides are 9 amino acids long (Fig. 1); however, peptides from 8 to 15 amino acids have been discovered. This is due to ‘bulging’ of the central section of the peptides, which allows the peptide to still fit within the binding groove (Fremont et al. 1992; Guo et al. 1992; Tynan et al. 2005). Furthermore, it has been shown that some pMHC-class I complexes are more immunogenic that others. Prediction models show that the amino acid position of a presented peptide is an important factor in this immunogenicity, where amino acids with large aromatic side chains may be better recognised by T-cells (Calis et al. 2013).

#### Peptide binding to MHC-class II

The MHC-class II epitope binding site consists of a groove and several pockets; X-ray crystallographic studies have shown these structures are provided by a β-sheet and two α-helices (Stern et al. 1994; Zhu et al. 2003). Unlike MHC-class I, the peptides that bind to MHC-class II proteins are not limited by the size of the binding groove, due to the MHC-class II peptide binding groove being open at both ends, allowing the binding of peptides up to 30 amino acids (Nelson and Fremont 1999). This results in different numbered positions within the peptide being able to bind to the anchor residues of MHC-class II molecules and makes residue motifs, rather than anchor positions important in defining and predicting peptides with optimal binding.

### MHC-class I/TCR interaction

The T-cell receptor is composed of two membrane anchored polypeptides, α and β that each contain one constant(C) and one variable domain. It is these hypervariable loops that recognise the pMHC complex displayed by cells. The pMHC complexes must form an immunological synapse with a TCR on T-cell in order to induce an immune response, which is dependent on additional co-stimulation and secretion of immunostimulatory cytokines by the activated T-cells to be sustained (Grakoui et al. 1999; Monks et al. 1998; Smith-Garvin et al. 2009; reviewed in Smith-Garvin et al. 2009). Cross-reactivity between TCR and pMHC recognition is likely given the number of possible combinations, which is an important consideration when manipulating TCR for immunotherapeutic purposes (Tan et al. 2015). Large-scale detection of antigen-specific T-cells is now possible using peptide MHC multimer technology (Bentzen et al. 2016; Luimstra et al. 2018), and the production of chimeric antigen receptors (Sharma and Kranz 2016), adoptive transfer of neoepitope reactive T-cells (Prickett et al. 2016) and TCR gene therapy (Blankenstein et al. 2015; Kato et al. 2018; Linnemann et al. 2014) have been shown to be promising treatment modalities utilising in silico prediction models.

## Types of cancer antigens

There are 3 broad classifications of tumour antigens that can be recognised as immune targets by T-cells: (a) cancer testis antigens (CT), (b) tumour-associated antigens (TA), and (c) tumour-associated antigens (TAA), including viral antigens (White et al. 2014) [e.g. human papilloma virus on cervical or oropharyngeal cancers (Gillison et al. 2000; Walboomers et al. 1999)] and neoantigens. This review will focus on those arising from genomic changes within cells as part of the tumourigenic process.

### CT antigens

CT antigens are a family of tumour-associated epitopes expressed on human tumours, but not on other tissues except for testis and placenta. Epigenetic alteration(s) appear to be the main mechanism regulating CT expression both in normal and neoplastic cells (Karpf and Jones 2002; Zendman et al. 2003). These epigenetic modifications allow for tissue-specific expression of transcripts in differentiated tissues and during development (Baylin and Jones 2011). The first description of epigenetic control of a CT antigen was of MAGE-1 promoter hypomethylation (De Smet et al. 1996, 1999), where demethylation removes the expression silencing mark. Since then, DNA methylation and histone post-translational modifications have been shown to be the most commonly employed mechanism controlling re-expression of the genes encoding CT antigens in tumour cells (Fratta et al. 2011; Siebenkas et al. 2017), reviewed in (Akers et al. 2010). Examples include NY-ESO-1 (Gnjatic et al. 2006) and members of the MAGE family (Chomez et al. 2001); a comprehensive list and evaluation of CT antigens are available at http://www.cta.lncc.br/ (Ludwig Institute for Cancer Research).

CT antigens represent promising therapeutic targets due to the following factors: (a) outside of tumour cells, expression is limited to germ cells (Chen et al. 2009; dos Santos et al. 2000; Greve et al. 2014; Hofmann et al. 2008; Sahin et al. 1998; Zendman et al. 2001); (b) epitopes from CT antigens are not displayed in the testis, as those cells do not express MHC-class I (Fiszer and Kurpisz 1998); (c) as the immune system has not interacted with the CT proteins, it is capable of being recognised as ‘non-self’ (Kalejs and Erenpreisa 2005). Further, immune responses to CT antigens are frequently observed in cancer patients (Akcakanat et al. 2004; Ayyoub et al. 2003; Milne et al. 2008; Qian et al. 2004; Tsuji et al. 2009; Wang et al. 2004) and there is an association between CT antigen expression and activity of tumour immune infiltrates (Rooney et al. 2015).

### TA antigens

TA antigens are overexpressed by cancer cells and comprise a large group that broadly can encompass any protein found at increase levels compared with normal tissue. They can be classified as (a) differentiation antigens, which are normal proteins overexpressed as a consequence of the tumourigenic proliferation of cells of a specific function [e.g. pigment production genes, such as tyrosinase in melanomas (Brichard et al. 1993) and the B-cell lineage-specific CD19 (Wang et al. 2012)]; or (b) overexpressed antigens, which are proteins that are minimally expressed by healthy, normal tissues, but are constitutively overexpressed by tumours as part of their malignant phenotype [e.g. PRAME (Kessler et al. 2001), p53 (Barfoed et al. 2000) and ERBB2 in breast cancer (Ellsworth et al. 2008)]. A comprehensive list of these antigens is available in an online database: https://caped.icp.ucl.ac.be/Peptide/list.

As TA antigens are derived from proteins that are overexpressed in a relatively high proportion of a given tumour type, as well as across different cancers, they represent attractive targets for the development of immunotherapy. While a number of antigenic peptides have been reported where immunoreactivity is observed (Vigneron et al. 2013), their use as an immunotherapy target is not devoid of risk. As they are expressed in normal tissue, TA antigens are more likely to have induced immunological tolerance and are less likely to stimulate effective anti-tumour immune responses (Cloosen et al. 2007; Yu et al. 2004).

### Types of neoantigen

Neoantigens can arise from any genomic mutation altering protein sequence, including non-synonymous mutations (e.g. Lennerz et al. 2005; Pritchard et al. 2015a), retained introns (e.g. Lupetti et al. 1998), post-translational modification that alters amino acid (e.g. Skipper et al. 1996), gene fusions (e.g. Chang et al. 2017) and frameshift in/del variants (e.g. Inderberg et al. 2017; Linnebacher et al. 2001). Next-generation sequencing (NGS) can be used to identify each of these types of variants, except for post-translational modification, which relies on techniques such as mass spectrometry. As the genomic variations are specific to cancer cells and are not present in the germline, they are not subject to central and peripheral tolerance. First identified in murine models (De Plaen et al. 1988; Monach et al. 1995), neoantigens have subsequently been shown to illicit an immune response capable of clearing tumour (Lennerz et al. 2005; Segal et al. 2008).

## Immunotherapeutic potential of cancer antigens

While the above-mentioned tumour antigens have been shown to elicit a robust immune response using autologous and donor in vitro testing (e.g. Jager et al. 1998; Knuth et al. 1984; Lennerz et al. 2005; Murray et al. 1992; Pritchard et al. 2015a; van der Bruggen et al. 1991; Vella et al. 2009), the translation of this to clinical application has largely resulted in low overall response rates (Ilyas and Yang 2015; Neller et al. 2008), with some notable exceptions (e.g. Bollard et al. 2014; Roskrow et al. 1998; Tran et al. 2014, 2016; Zacharakis et al. 2018). As neoantigens are more likely to be different to ‘self’ than CT or TA antigens, the affinity of the TCR recognising the HLA-bound peptide and subsequent strength of the immune response tends to be stronger (Aleksic et al. 2012; Tan et al. 2015). Furthermore, as TA antigens are produced by normal cells and CT antigens may have significant homology to proteins produced on normal cells, their use poses a risk of autoimmunity [e.g. destruction of normal melanocytes in the skin, eye and ear (Johnson et al. 2009)], which can sometimes have devastating unintended consequences to the patient (e.g. Cameron et al. 2013; Morgan et al. 2010, 2013). The more successful clinical approaches have therefore tended to be using viral (e.g. Bollard et al. 2014; Heslop et al. 2010; Louis et al. 2010; Schuessler et al. 2014; Smith et al. 2017) and neoantigen targeting (Sahin et al. 2017; Tran et al. 2014, 2016).

This review will now focus on the neoantigens; for more general overviews of the processes involved in antigen processing and targeting these antigens immunotherapeutically, the following reviews are recommended: Coulie et al. (2014), Lu and Robbins (2016), Pritchard (2018), Tashiro and Brenner (2017).

## Identification of neoantigens

Some tumours are more highly mutated than others (Lawrence et al. 2013), resulting in a difference in likelihood of immunogenic neoantigen production between cancer types (Schumacher and Schreiber 2015). As melanoma is among the most genomically mutated tumours, with a high number of non-synonymous single-nucleotide variants (Hayward et al. 2017; Lawrence et al. 2013), it is one of the most frequently studied (e.g. Lauss et al. 2017; Lennerz et al. 2005; Ott et al. 2017; Pasetto et al. 2016; Pritchard et al. 2015a; Snyder et al. 2014; Stronen et al. 2016; van Rooij et al. 2013; Verdegaal et al. 2016). Prior to the advent of massively parallel NGS, the method to identify neoantigens was by labour-intensive individual cDNA library screening (e.g. as performed in Lennerz et al. 2005) and as a result, the number of identified and studied neoantigens was fairly low. Once whole exome/genome sequencing became a routine technique (reviewed in Goodwin et al. 2016), the ability to identify tumour-specific genetic mutations altering the protein coding regions became rapid and high throughput, facilitating neoantigen prediction. Mass spectrometry can also be used to identify peptides bound to the MHC molecules on the surface of cells (Bassani-Sternberg et al. 2016; Freudenmann et al. 2018; Hunt et al. 2007; Mommen et al. 2014; Pritchard et al. 2015b; Purcell 2004; Purcell and Gorman 2004; Tan et al. 2011). Despite these advances, neoantigens are only rarely detected on cancer cells using mass spectrometry and further studies are required to discover why this is the case (Abelin et al. 2017; Bassani-Sternberg et al. 2016; Carreno et al. 2015; Gloger et al. 2016; Gubin et al. 2014; Hogan et al. 1998; Jarmalavicius et al. 2012; Pritchard et al. 2015b; Yadav et al. 2014).

The majority of recent studies have therefore focused on a forward approach of performing NGS on germline and tumour DNA to identify protein altering mutations that are specific to the cancer cells, followed by epitope prediction via in silico algorithms, as detailed in the following sections.

### Prediction of neoantigen(s) in the era of next-generation sequencing technology

The prediction of neoantigens relies on the in silico processing of genomic data and requires knowledge of the donors HLA type, tumour mRNA expression, germline DNA and tumour DNA. The tumour mRNA expression data such as whole genome microarrays (e.g. Pritchard et al. 2015a) or RNA-seq (e.g. Karasaki et al. 2017; van Rooij et al. 2013) are overlaid on tumour-specific cancer mutation information, to identify variants in transcribed genes. These variants are then run through epitope prediction algorithms, to identify peptide sequences that potentially bind to individual-specific HLA-alleles, which requires the amino acid sequence to be translated from the surrounding genetic sequence. A confounding factor is population polymorphism, which if in phase with the somatic variant may additionally alter amino acids from the reference sequence. There are many epitope prediction algorithms available, including SYFPEITHI (Schuler et al. 2007), RANKPEP (Reche et al. 2002), NetMHCpan (Jurtz et al. 2017), NetMHCcons (Karosiene et al. 2012), PickPocket (Zhang et al. 2009), MHCflurry (in pre-print, 10.1101/174243), ANN (Singh and Mishra 2008) and SMM (Peters and Sette 2005). These algorithms employ different prediction models but have all been trained using characterised epitope/MHC combinations, resulting in the prediction of the likelihood of short peptide sequences binding to a given HLA-allele. Bioinformatic pipelines have been created that use whole genome/exome sequencing data and integrate the analysis to include HLA-allele typing, mRNA expression data, peptide processing prediction and HLA-allele binding for the wildtype and mutated peptide. These include pVAC-seq (Hundal et al. 2016), MuPeXi (Bjerregaard et al. 2017a), Cloudneo (Bais et al. 2017) and TIminer (Tappeiner et al. 2017). Immune evasion and editing can be a limitation to specific T-cell immunotherapy, resulting either in failure to initiate tumour clearance or acquired resistance to therapy. This includes loss of HLA expression by chromosomal 6 loss of heterozygosity (LOH) or the down-regulation of support molecules by various methods (e.g. Anagnostou et al. 2017; Chowell et al. 2018; Schrors et al. 2017). To partially address this issue, the computational tool LOHHLA (loss of heterozygosity in human leukocyte antigen) allows allele-specific copy number estimation of the HLA locus from next-generation sequencing data (McGranahan et al. 2017). The refinement of neoantigen prediction by a combination of the above methods will improve the likelihood of immunogenic neoantigens being identified, which has the potential to improve immunotherapeutic approaches targeting neoantigens.

#### Prediction of MHC-class I compared to MHC-class II neoantigen binding

As the majority of studies identifying peptides that bind to MHC molecules have focused on those recognised by cytotoxic CD8^+^ T-cells, the prediction of antigen binding to MHC-class I molecules is the most studied. This is influenced both by the function of the cytotoxic CD8^+^ T-cells in directly triggering programmed cell death and the way in which the peptide fits into the MHC-class I binding groove, which makes the prediction of this binding more amenable to machine learning. Specifically, as previously described, the lack of definitive N- and C-anchor points within the MHC-class II binding groove makes the prediction of peptides that may bind more difficult (Wang et al. 2008).

While the role of the T_H1_ subset of CD4^+^ T-cells in priming, supporting, recruiting and proliferation of CD8^+^ T-cells is well established, CD4^+^ T-cells recognising immunoreactive MHC-class II-restricted neoantigens have also been described (e.g. Linnemann et al. 2015; Pieper et al. 1999; Tran et al. 2015; Veatch et al. 2018; Wang et al. 1999; reviewed in Sun et al. 2017). The number of MHC-class II-restricted epitopes catalogued is substantially lower compared to MHC-class I. The number of unique peptides of different lengths that are classified as binding to MHC-class I (*n* = 229,035) and MHC-class II (*n* = 54,606) in the IEDB database (Vita et al. 2010) https://www.iedb.org/ is shown in Fig. 1, with the most frequently identified peptide length found to bind to MHC-class I being 9 amino acids long and 15 amino acids long for MHC-class II.

##### MHC-class I peptide consensus binding sequences

**Fig. 2:**
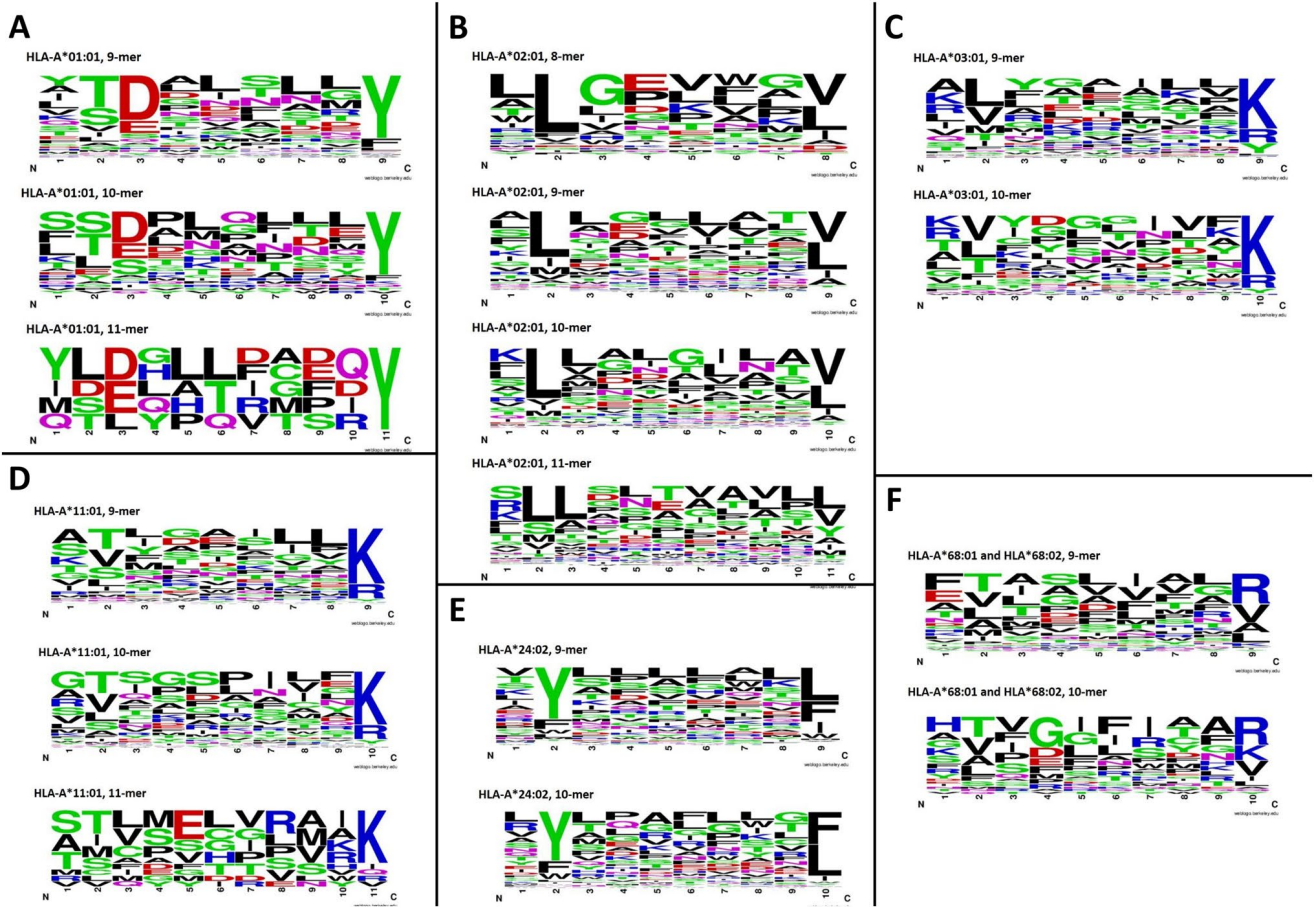
Consensus logos motifs for peptides binding to selected MHC-class I HLA-A alleles. The logo motifs were created using WebLogo (http://www.weblogo.berkeley.edu/logo.cgi). Peptides for each HLA type were grouped by peptide length and input to WebLogo, with default settings polar amino acids (G, S, T, Y, C, Q, N) are green, basic (K, R, H) blue, acidic (D, E) red and hydrophobic (A, V, L, I, P, W, F, M) amino acids are black. Where sufficient peptides are present (*n* > 10) for each peptide length, a consensus logo motif was created. **a** HLA-A*01:01 (*n* = 147); **b** HLA-A*02:01 (*n* = 2536); **c** HLA-A*03:01 (*n* = 213); **d** HLA-A*11:01 (*n* = 222); **e** HLA-A*24:02 (*n* = 282) **f** HLA-A*68:01 and HLA*68:02 (combined *n* = 51). (Color figure online)

**Fig. 3:**
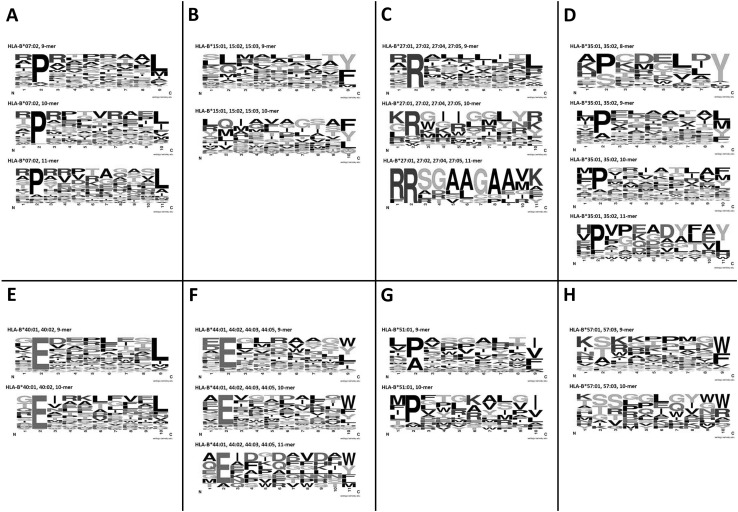
Consensus logos motifs for peptides binding to selected MHC-class I HLA-B alleles. The logo motifs were created using WebLogo (http://www.weblogo.berkeley.edu/logo.cgi). The overall height of the stack indicates the sequence conservation at that position, while the height of symbols within the stack indicates the relative frequency of each amino or nucleic acid at that position. Peptides for each HLA type were grouped by peptide length and input to WebLogo, with default settings polar amino acids (G, S, T, Y, C, Q, N) are green, basic (K, R, H) blue, acidic (D, E) red and hydrophobic (A, V, L, I, P, W, F, M) amino acids are black. Where sufficient peptides are present (*n* > 50) for each peptide length, a consensus logo motif was created. **a** HLA-B*07:02 (*n* = 337); **b** HLA-B*15:01, B*15:02, B*15:03 (combined *n* = 224); **c** HLA-B*27:01, B*27:02, B*27:04, B*27:05 (combined *n* = 114); **d** HLA-B*35:01, B*35:02 (combined *n* = 296); **e** HLA-B*40:01, B*40:02 (combined *n* = 94); **f** HLA-B*44:01, B*44:02, B*44:03 (combined *n* = 118); **g** HLA-B*51:01 (*n* = 100); **h** HLA-B*57:01, B*57:03 (combined *n* = 80). (Color figure online)

In MHC-class I, the population polymorphisms that dictate HLA-subtype can affect the peptide binding groove, including the anchor residues at the N- and C-terminals, resulting in different peptide epitopes preferentially binding depending on amino acid sequence. Once sufficient peptides binding to different HLA subtypes have been characterised, consistent motifs within these amino acid sequences can be identified. Figure 2 shows the consensus motif for six HLA-A subtypes and Fig. 3 shows the motifs for eight HLA-B subtypes. The peptides used to create these motifs were selected from the IEDB, based on those with a sufficient number of peptides to assess (*n* ≥ 50) and targeting those that have been shown by functional experiment to elicit a T-cell response. These figures illustrate that there are clear anchor motifs at the C- (HLA-A, Fig. 2) and N- (HLA-B, Fig. 3) terminals for the selected HLA subtypes. Additionally, for the more common HLA types (such as HLA-A*01:01 and HLA-A*02:01, Fig. 2 and HLA-B*07:02 and HLA-B*40:02, Fig. 3), consensus binding amino acids are emerging at the N- and C-terminals, respectively. The simple (single amino acid binding) and complex (e.g. common properties of several amino acids) types of common features visible in the consensus logo motifs in Figs. 2 and 3 inform the epitope binding prediction algorithms described in “Types of neoantigen” section.

#### Neoantigen-specific T-cell receptors

Antigen-specific TCR gene transfer via patient-derived T-cells provides the opportunity to break self-tolerance and improve the affinity by which cognate TCR binding to ‘self’-derived epitopes can occur. TCR gene therapy has been a successful means of targeting CT and TA antigens (e.g. Morgan et al. 2006; Robbins et al. 2011); however, off target reactivity has been an issue for these therapies, particularly if cross-reactivity between the modified TCR and other ‘self’ antigen occurs (e.g. Cameron et al. 2013; Tan et al. 2015).

As neoantigens occur only within tumour tissue, they do not induce central tolerance and neoantigen-specific TCRs may therefore be more specific and have a higher affinity than TCR targeted to non-mutated antigens. Preclinical studies have shown that the adoptive transfer of neoantigen-specific TCR engineered T-cells can be effective against solid tumours (Bendle et al. 2010; Boulter et al. 2003); however, in practice, unexpected cross-reactivities have hampered their use (e.g. Morgan et al. 2010, 2013), highlighting the need for careful manipulation of the immune system. NGS technologies are enabling comprehensive description of patient-specific TCR repertoires, allowing the identification of the frequencies of unique TCR clonotypes among TILs (reviewed in Rosati et al. 2017). Multiple technologies have emerged for the isolation of TCR genes, allowing the rapid identification of large TCR libraries from intratumoural T-cells. This has therefore facilitated the careful assessment of antigen specificity of intratumoral TCRs independent from primary material. Furthermore, the availability of such TCR gene libraries may facilitate efforts to locate target epitopes within the cancer anti-genome (Hanson et al. 2016; Howie et al. 2015; Kato et al. 2018; Kwong et al. 2009).

#### Accuracy of epitope prediction in identifying immunoreactive antigens

**Fig. 4:**
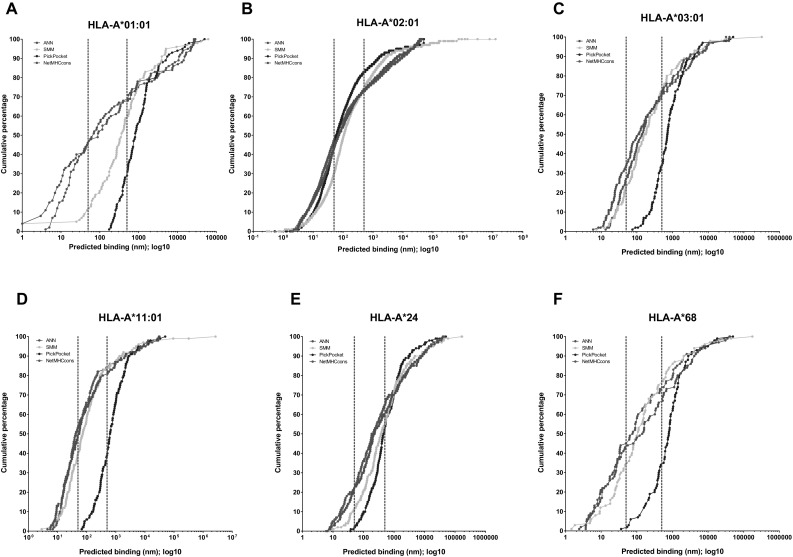
Cumulative percentage plots of epitope prediction score for HLA-A subtypes. Using the epitope dataset present in the IEDB with proven T-cell stimulating ability and known HLA-A subtype (total *n* = 3451), four prediction algorithms present in IEDB (ANN, SMM, NetMHCcons and PickPocket) were used to provide epitope prediction scores. The cumulative percentage plots were created to show the proportion of scores that fit within the traditional binding scores for ‘strong’ (< 50 nM) and ‘weak’ (< 500 nM) interactions. These values are indicated by the red dotted lines. **a** HLA-A*01:01 (*n* = 147); **b** HLA-A*02:01 (*n* = 2536); **c** HLA-A*03:01 (*n* = 213); **d** HLA-A*11:01 (*n* = 222); **e** HLA-A*24 (*n* = 282); **f** HLA-A*68 (combined *n* = 51)

As each of the algorithms described in the “Types of neoantigen” section are influenced by the training data set, the less common HLA-alleles tend to have less peptides present, resulting in less confident (higher) binding scores. This means these less common HLA subtypes tend to be consistently predicted to bind less well, compared to the more common HLA subtypes (e.g. as assessed in Pritchard et al. 2015a, b). These analyses are extended in Fig. 4, using the epitope dataset stored in the IEDB with proven T-cell stimulating ability (total *n* = 3632), which were run through four prediction algorithms present in IEDB (ANN, SMM, NetMHCcons and PickPocket). The cumulative percentage plots illustrate that all four prediction algorithms have a similar prediction profile for the most common HLA-A*02 allele; however, the other five HLA-A alleles examined show different abilities of the algorithms to predict similar results. Additionally, for the most common HLA-A*02 allele, just under 50% of the binding scores are < 50 nM, traditionally defined as ‘strong’ binders, while between 70 and 80% of the scores are < 500 nM, traditionally defined as ‘weak’ binders. This pattern is similar for the other HLA-A alleles assessed (HLA-A*01, HLA-A*03, HLA-A*11, HLA-A*24 and HLA-A*68) for ANN and NetMHCcons; however, SMM and PickPocket are more conservative predictors for these less common alleles, with between 0 and 50% of all peptides classified as ‘weak’ binders.

**Fig. 5:**
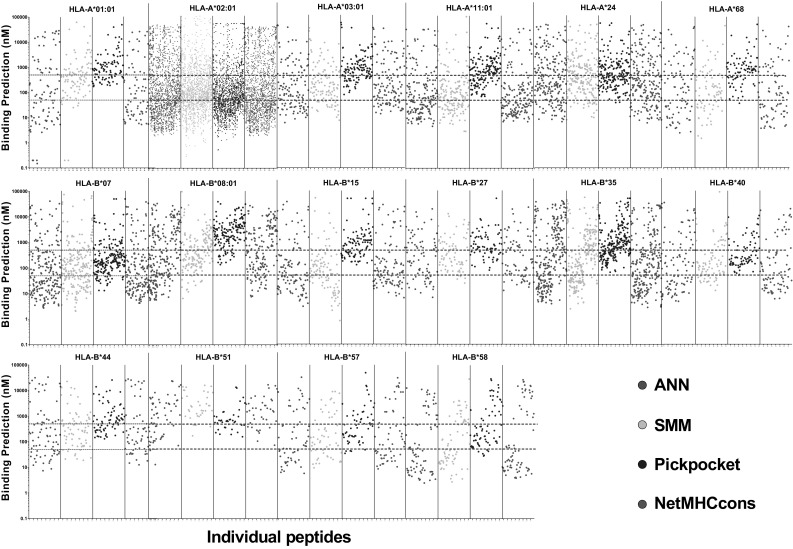
Individual peptide scores plotted for each prediction algorithm. Using the IEDB MHC-class I T-cell activating epitopes that bind to defined HLA-A or HLA-B alleles (*n* = 4814), four prediction algorithms present in IEDB (ANN, SMM, NetMHCcons and PickPocket) were used to provide epitope prediction scores. These are individually plotted to visualise the number of available peptides to assess and the performance of each algorithm against each other and the traditional binding scores for ‘strong’ (< 50 nM) and ‘weak’ (< 500 nM) interactions. These values are indicated by the red dotted lines. (Color figure online)

A further analysis using the IEDB MHC-class I T-cell activating epitopes that bind to both HLA-A and HLA-B alleles (*n* = 5510) has been carried out. These were run through the same four prediction algorithms as above and then all binding scores graphed (Fig. 5). These graphs illustrate that using the aforementioned standard thresholds to indicate “strong” and “weak” binding, as indicated by the red dotted lines, is likely to miss a large number of epitopes that have been proven to elicit T-cell responses; this is particularly striking in the less common HLA subtypes. These data also further illustrate the differences in binding prediction scores between the different algorithms assessed. Given that these analyses were carried out using functionally characterised immunogenic peptides, an interpretation of these data might be that those algorithms with a large number of prediction scores > 1000 nM are less robust predictors for those HLA-alleles.

Data from repositories such as IEDB analysed in a manner such as performed here can be used to aid the selection of the most informative algorithms for the HLA subtypes of interest. Indeed, it has been shown that by analysing immunogenic neopeptides and peptides from the same studies that do not elicit a T-cell response, prediction of binding patterns may be improved (Bjerregaard et al. 2017b). Further, as more studies identify and functionally examine the peptides binding to HLA-alleles are carried out, the more robust these analyses will become; the current interest in this field is significantly increasing available data.

### Use of neoantigen prediction in practice

A large number of published studies have assessed the ability of protein altering mutations in cancer cells to form neoantigens using in silico tools (e.g. Balachandran et al. 2017; Brown et al. 2014; Gros et al. 2016; Hodges et al. 2017; Lauss et al. 2017; Linnemann et al. 2015; Luksza et al. 2017; Matsushita et al. 2012; McGranahan et al. 2016; Ock et al. 2017; Pritchard et al. 2015a; Rizvi et al. 2015; Robbins et al. 2013; Snyder et al. 2014; Tran et al. 2016; Van Allen et al. 2015; van Rooij et al. 2013; Verdegaal et al. 2016). While these various tools have an important role to play in the prediction of immunogenic antigens, the majority of the identified epitopes do not initiate an immune response (e.g. as assessed in Gros et al. 2016; Linnemann et al. 2015; McGranahan et al. 2016; Pritchard et al. 2015a; Robbins et al. 2013; Schmidt et al. 2017; Snyder et al. 2014; van Rooij et al. 2013). The possible reasons behind suboptimal immunogenicity of peptide vaccines for cancer are reviewed by Kumai et al. (2017).

A recent study specifically tackled the question of how many predicted epitopes are capable of eliciting an immune response, using the CEF peptide pool (consisting of 32 individual peptides from Cytomegalo-, Epstein–Barr-, and Influenza viruses) and a panel of 42 HLA typed HLA-A*02:01 positive individuals. There were 241 different peptide stimulating CD8^+^ responses predicted, on the basis of individual HLA-typing. Broadly, 51% of these predictions stimulated a response, of varying strengths, with only 15% occurring in the high frequency range (> 100 spots/400,000 PBMC) and 17% in mid-frequency (> 10 spots/400,000 PBMC) and 19% at the detection limit (1 spot/400,000 PBMC). Fifty-seven unpredicted responses were seen, meaning that of all the responses detected, 68% were predicted and 32% were not. In total, 49% of the predicted peptides were not detectably targeted by CD8^+^ cells (Moldovan et al. 2016). These data are intriguing on several levels. The first is that the CEF peptides are among the most studied immune responses and that both predicted and unpredicted CD8^+^ T-cell response after exposure to these peptides are observed indicates there are still significant aspects of epitope prediction that are not yet achieved. The second is that despite prediction in these well characterised antigens, a large proportion did not elicit any detectable immune response. Finally, despite detecting a CD8^+^ T-cell response to approximately half of the epitopes predicted, only a fraction were a dominant response. Extrapolation of these data to that observed for neoantigen prediction in studies that do not test the potential epitopes for immune cell recognition should cause a pause for thought in how these data are reported and presented.

## Testing of neoantigen immunogenicity

It is clear from the published studies that while the data from the prediction algorithms can be used to inform on the potential epitopes created, there still needs to be laboratory testing for immunogenicity of these epitopes. There are a number of methods by which this can be performed, including screening of the predicted peptides across mixed lymphocyte-tumour culture (MLTC) (e.g. Lennerz et al. 2005; Pritchard et al. 2015a), exposure of tandem mini-genes (e.g. Gros et al. 2016; Lu et al. 2014; Mennonna et al. 2017; Tran et al. 2014, 2015) or pMHC multimers (e.g. Cohen et al. 2015; Stronen et al. 2016; van Rooij et al. 2013) to immune cells, and the pulsing of putative peptides with antigen presentation cells (such as dendritic cells or B-cells) and co-culture with T-cells, followed by T-cell exposure to predicted peptide pools (e.g. Rajasagi et al. 2014). These approaches can identify existing memory T-cell immune responses in patients, or reactive naïve T-cells in patients/donors, both of which have potential clinical utility.

## Immunotherapeutic potential of neoantigens

Neoantigens have been shown to contribute to the success of various immunotherapies, including the checkpoint inhibitors targeting PD-1/PD-L1/CTLA4 (e.g. Balachandran et al. 2017; Snyder et al. 2014; van Rooij et al. 2013) and other forms of immunotherapy, including dendritic cell vaccines [e.g. unpublished observations and Pritchard et al. 2015a, assessing patients from clinical trials (O’Rourke et al. 2003, 2007)] and adoptive T-cell transfer (e.g. Tran et al. 2016; Verdegaal et al. 2016). Additionally, as neoantigens are capable of stimulating tumour clearance (e.g. Zacharakis et al. 2018), there are currently a number of registered clinical trials that include combination of immunotherapies with a personalised neoantigen component (e.g. with anti-PD-1 checkpoint inhibition NCT02950766, renal cell carcinoma; NCT03199040, triple-negative breast cancer) and dendritic cell vaccine raised against defined neoantigens (e.g. NCT03300843, melanoma, gastrointestinal, breast, ovarian and pancreatic cancers, NCT03558945, pancreatic cancer) and adjuvant personalised neoantigen peptide vaccine, with the immunostimulant poly-ICLC (e.g. NCT02510950, glioblastoma and astrocytoma and NCT01970358, melanoma). Although it may be possible to target the more common ‘driver’ gene mutations such as BRAF (Sharkey et al. 2004; Somasundaram et al. 2006; Veatch et al. 2018), KRAS (Bergmann-Leitner et al. 1998; Shono et al. 2003), p53 (Ichiki et al. 2004) and NRAS (Linard et al. 2002), the required combination of non-synonymous variant and specific HLA-allele make neoantigens more likely to be a personalised therapy option.

As illustrated with examples above, the major limitation to the use of neoantigens in immunotherapy is the reliable personalised prediction of those that will have undergone proteasomal cleavage, transport to the ER, binding to the individuals HLA molecule and recognition by the T-cell receptor to stimulate an immune response capable of tumour clearance.

## Conclusion

The somatic mutations acquired by cancer cells can be recognised as ‘non-self’ by the immune system and are capable of inducing an immune response that can selectively target and remove tumour cells. There are a number of steps required in order for the peptides to be displayed to the immune system and each of these processes has optimal conditions under which they occur. Therefore, despite there being a large number of potential neoantigens in some cancers with high mutation burden, only a fraction are able to ultimately mount an immune response. With the improvement in molecular and in silico capabilities in recent years, the number of identified immunogenic neoantigens has substantially increased. As demonstrated here, the current methods do not consistently identify epitopes that are a priori known to mount an immune response. With a greater number of verified neoantigens, the better the ability of trained in silico prediction tools to reliably identify those that may have clinical utility. There is considerable potential in the use of neoantigens to treat patients, either alone or in combination with other immunotherapies and with continued advancements, these potentials will be realised.
